# Heavy-ion production of ^77^Br and ^76^Br

**DOI:** 10.1038/s41598-021-94922-x

**Published:** 2021-08-03

**Authors:** Sean R. McGuinness, John T. Wilkinson, Graham F. Peaslee

**Affiliations:** Department of Physics, University of Notre Dame, Notre Dame, IN 46556 USA

**Keywords:** Nuclear physics, Experimental nuclear physics

## Abstract

Many radioisotopes with potential medical applications are difficult to produce routinely, especially those on the proton-rich side of the valley of stability. Current production methods typically use light-ion (protons or deuteron) reactions on materials of similar mass to the target radioisotope, which limits the elemental target material available and may require the use of targets with poor thermal properties (as is the case for the production of radiobromine). These reactions may also create significant amounts of proton-rich decay products which require chemical separation from the desired product in a highly radioactive environment. A promising alternative method using heavy-ion fusion-evaporation reactions for the production of the medically relevant bromine radioisotopes ^76^Br (t_1/2_ = 16.2 h) and ^77^Br (t_1/2_ = 57.0 h) is presented. Heavy-ion beams of ^28^Si and ^16^O were used to bombard natural chromium and copper targets just above the Coulomb barrier at the University of Notre Dame's Nuclear Science Laboratory to produce these bromine and precursor radioisotopes by fusion-evaporation reactions. Production yields for these reactions were measured and compared to PACE4 calculations. In addition to using more robust targets for irradiation, a simple physical–chemical separation method is proposed that will lead to very high radiopurity yields. A summary of accelerator facility requirements needed for routine production of these radioisotopes is also presented.

## Introduction

Radioisotopes of bromine are uniquely suited for use in theranostic compounds (compounds which can be used for both therapeutic and diagnostic purposes). ^77^Br (t_1/2_ = 57.0 h) is an Auger-emitter with potential therapeutic uses, while ^76^Br (t_1/2_ = 16.2 h) is a positron-emitter with broad diagnostic utility. Both have been used extensively in various molecular formulations (^1,2^ and references therein), and can in principle be used in any compound in which radioiodine is used, with the benefit of a stronger bond to carbon and no dosimetric build-up in the thyroid in the event of in vivo dehalogenation^3,4^.

Current methods of production for both bromine radioisotopes use energic light-ion beams (typically protons or alpha particles) to bombard upon targets close to bromine on the periodic table. With proton beams, isotopically enriched selenium targets can be used to produce the desired bromine isotopes, but elemental selenium does not withstand irradiation well; the low boiling point, high vapor pressure and poor conductivity (electrical and thermal) ensure that any target will substantially degrade even with limited proton flux^5^. Targets using compounds of selenium and copper^6,7^, nickel^8,9^, zinc^7^ and cobalt^5^ have been attempted, but, while significantly better at withstanding irradiation than pure selenium targets, proton intensities must still be limited to 40µA, leading to limitations on the achievable yield of ^76/77^Br. In addition, target degradation ensures that new targets must be produced after using the target a limited number of times. Reported yields have been up to 10.6 GBq per 3-h irradiation (88 MBq∙μA^−1^∙h^−1^) for ^76^Br and 2.8 GBq per 3-h irradiation (23 MBq∙μA^−1^∙h^−1^) for ^77^Br^5^. With alpha beams, elemental arsenic targets have been used, with similar issues of target degradation. ^76^Br cannot be produced by this method without the co-production of an at least equal amount of ^77^Br. Yields of 9.1 MBq∙μA^−1^∙h^−1^ for ^77^Br have been reported from 30-min irradiations with 70 nA currents^10^. Taken together with radiobromine’s promising radiochemistry and in vivo applications, there is a clear need for alterative production methods for these promising radioisotopes.

## Heavy-ion fusion-evaporation reactions

This work demonstrates the feasibility of a different approach to produce ^76^Br and ^77^Br: symmetric heavy-ion reactions. Symmetric heavy-ion fusion-evaporation reactions have been proposed to produce other medically relevant radionuclides^11,12^ and have several advantages over proton or alpha spallation reactions. The most significant advantage is the isotopic yield specificity since the amount of energy lost through light charged particle evaporation restricts the number of resultant radionuclides formed in each reaction. More symmetric heavy ion entrance channels also increase the ability to produce proton-rich compound nuclei far from the valley of stability. Two promising systems were chosen for exploration into producing radiobromine: a ^28^Si beam bombarding a natural chromium target and an ^16^O beam bombarding natural copper.

### ^28^Si on ^nat^Cr

For the ^28^Si beam, the primary exit channel of interest is a (2p + n) fusion-evaporation reaction on ^52^Cr:

1 $$^{{{28}}} {\text{Si}} +\, ^{{{52}}}{\text{Cr}} \to\, ^{{{8}0}}{\text{Sr}}^* \to\, ^{{{77}}}{\text{Kr}} + {\text{2p}} + {\text{n}}$$

**Figure 1:**
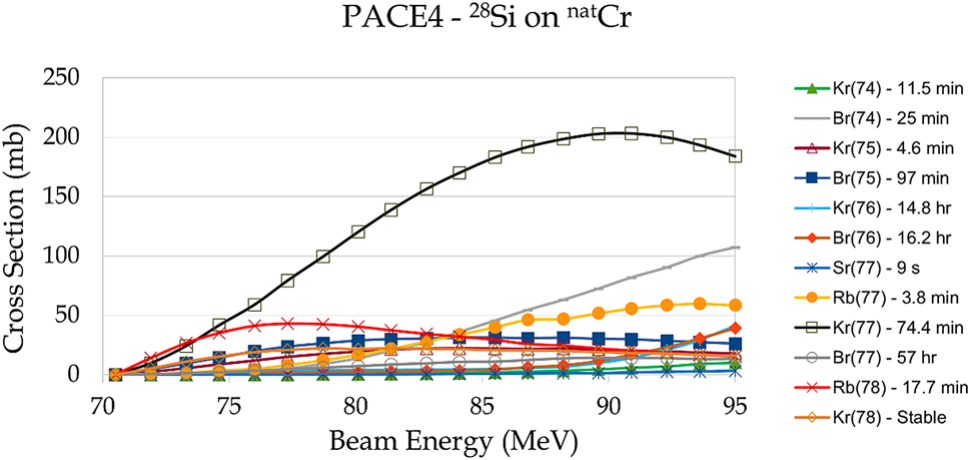
PACE4 predicted cross sections for ^77^Br, precursors and contaminants as a function of laboratory bombarding energy for the ^28^Si + ^nat^Cr reaction. The dominant products are ^77^Rb and ^77^Kr, which decay to ^77^Br. The mass-76 isobars are not strongly produced.

^52^Cr makes up 84% of natural chromium, so isotopically enriched targets are not required. Reactions on the other chromium isotopes have similar products, although with reduced production of the mass-77 isobars. The only strongly produced contaminant solely from the non-^52^Cr isotopes is short-lived ^74^Br (t_1/2_ = 25 min), which would not interfere with routine production of ^77^Br. The PACE4^13,14^ fusion-evaporation code was used to predict production cross-sections. Calculations were performed at 10,000 cascades per energy to minimize simulation error, which is on the order of one millibarn. The cross-sections for various relevant radionuclides (bromine and radionuclides which decay to bromine isotopes) are presented in Fig. 1 as a function of laboratory bombarding energy. Production of the mass-77 isobar chain dominates at all energies.

### ^16^O on ^nat^Cu

For the oxygen on copper system, the reactions of interest are multi-nucleon-evaporation reactions on both ^63^Cu and ^65^Cu:

2 $$^{{{16}}} {\text{O}} + \,^{{{63}}}{\text{Cu}} \to\, ^{{{79}}}{\text{Rb}}^* \to\,^{{{77}}}{\text{Kr}} + {\text{p}} + {\text{n}}$$

3 $$^{{{16}}} {\text{O}} +\,^{{{63}}}{\text{Cu}} \to\, ^{{{79}}}{\text{Rb}}^* \to\,^{{{76}}}{\text{Kr}} + {\text{p}} + {\text{2n}}$$

4 $$^{{{16}}} {\text{O}} +\,^{{{63}}}{\text{Cu}} \to\, ^{{{79}}}{\text{Rb}}^* \to\,^{{{76}}}{\text{Br}} + {\text{2p}} + {\text{2n}}$$

5 $$^{{{16}}} {\text{O}} +\,^{{{65}}}{\text{Cu}} \to\, ^{{{81}}}{\text{Rb}}^* \to\,^{{{77}}}{\text{Kr}} + {\text{p}} + {\text{3n}}$$

6 $$^{{{16}}} {\text{O}} +\,^{{{65}}}{\text{Cu}} \to \,^{{{81}}}{\text{Rb}}^* \to\,^{{{76}}}{\text{Br}} + {\text{2p}} + {\text{3n}}$$

**Figure 2:**
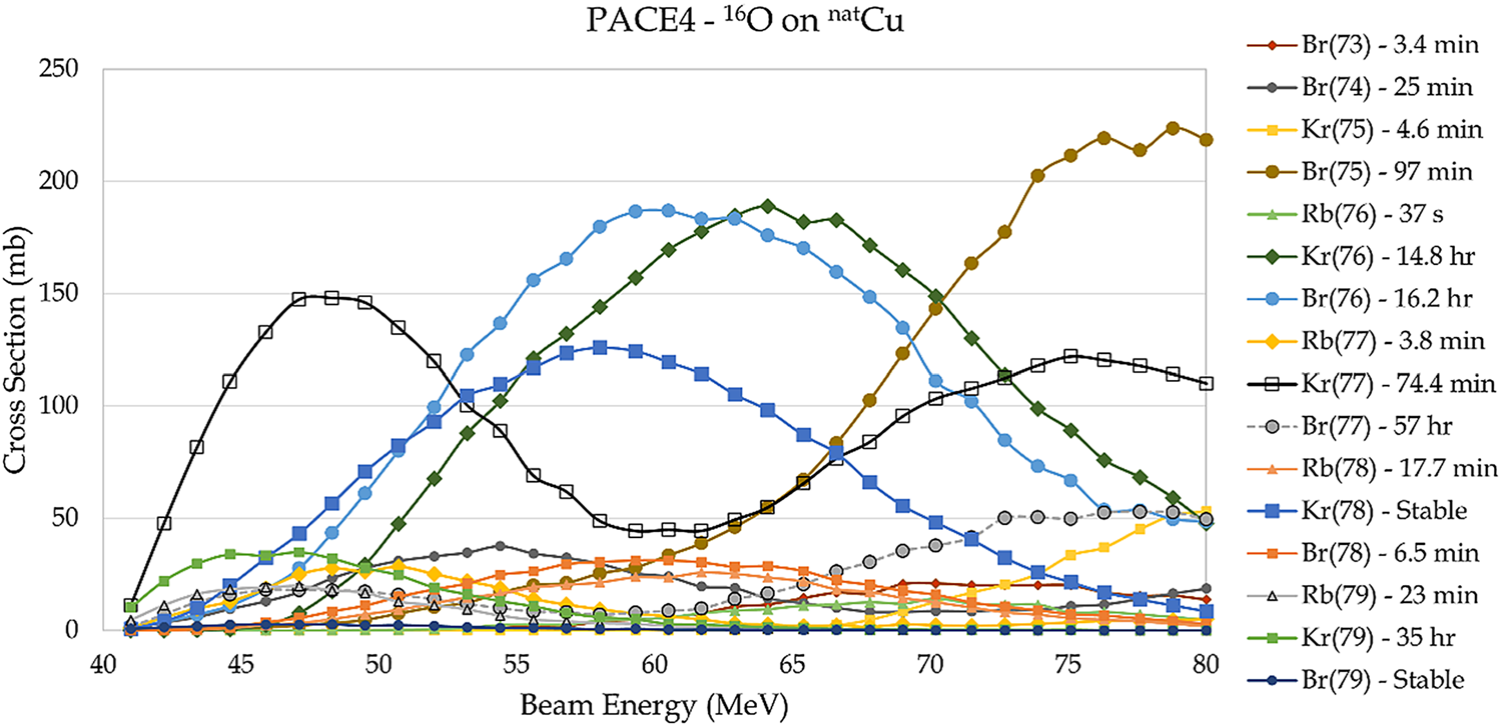
PACE4 predicted cross sections for ^16^O on ^nat^Cu as a function of laboratory bombarding energy. All relevant isotopes are shown with their respective half-lives. The double-humped structure is the result of Q-value differences between reactions on ^63^Cu and ^65^Cu. The mass 77 and 76 decay chains are both produced strongly.

**Figure 3:**
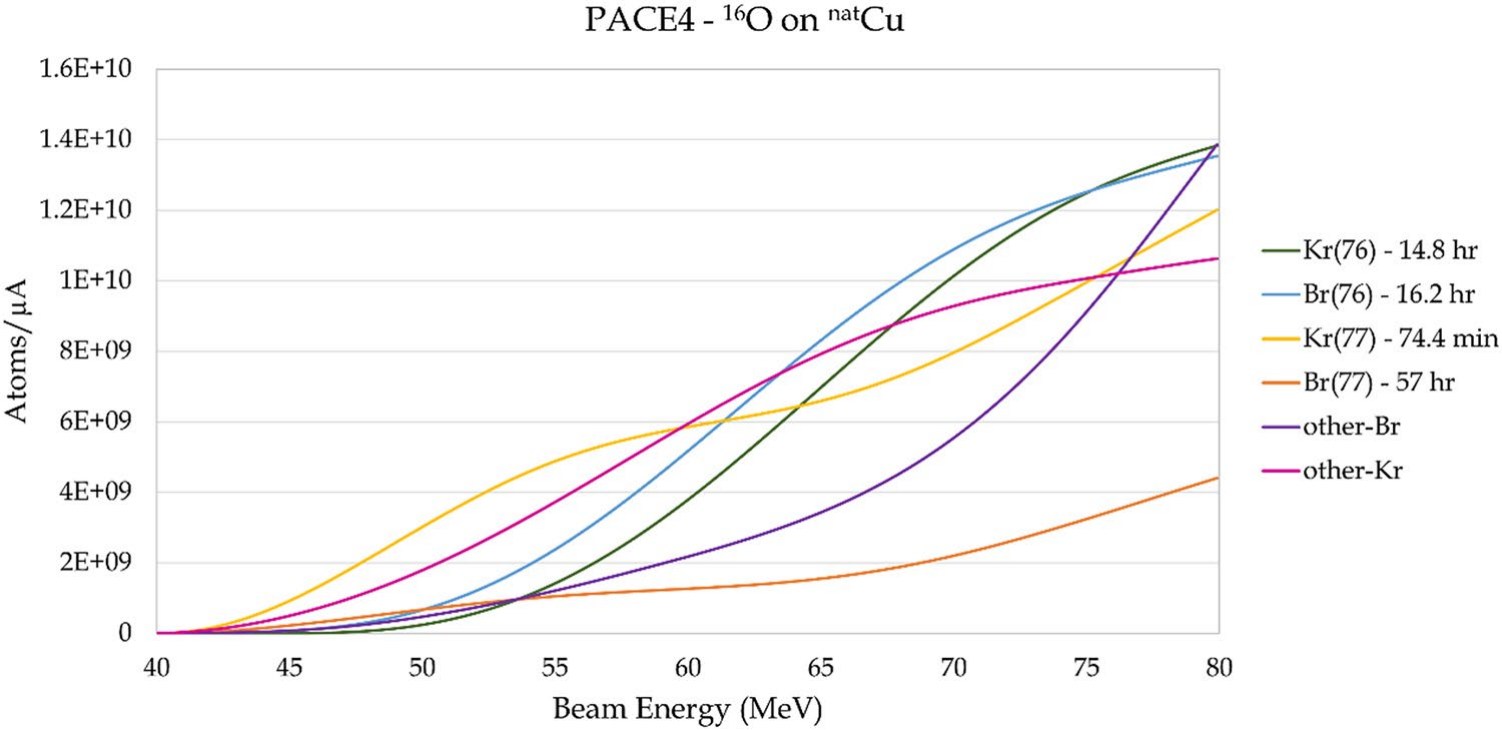
PACE4 predicted yields for a 20-min run of ^16^O on ^nat^Cu as a function of laboratory bombarding energy. The mass-76 and mass-77 isobar chains are shown explicitly, while all contaminant isotopes are shown summed together.

These reactions are all available on natural copper, and again isotopically enriched targets are not required. If a mixed source of ^76/77^Br is not desirable, then careful energy selection is required to favor the production of one isotope over the other. In that case, isotopically pure targets of either ^63^Cu or ^65^Cu could be advantageous. For the purposes of this proof-of-principle study, a mixed source was studied. The Stopping Range of Ions in Matter (SRIM^15^) program was used to calculate the energy-loss-profile for the beam in the target. The energy loss was then used to calculate the beam energy as a function of target penetration depth, which was then combined with the PACE4 cross sections and a delivered-current profile to produce predicted yields. Figures 2 and 3 show the predicted cross sections and yields for the production of various Rb, Kr and Br isotopes from the ^16^O bombardment of ^nat^Cu as a function of laboratory bombarding energy (Figs. 10 and 11 in the supplemental section present similar plots for isotopically pure ^63/65^Cu targets). The mass-77 and mass-76 isobars can both be strongly produced.

### Separations

**Figure 4:**
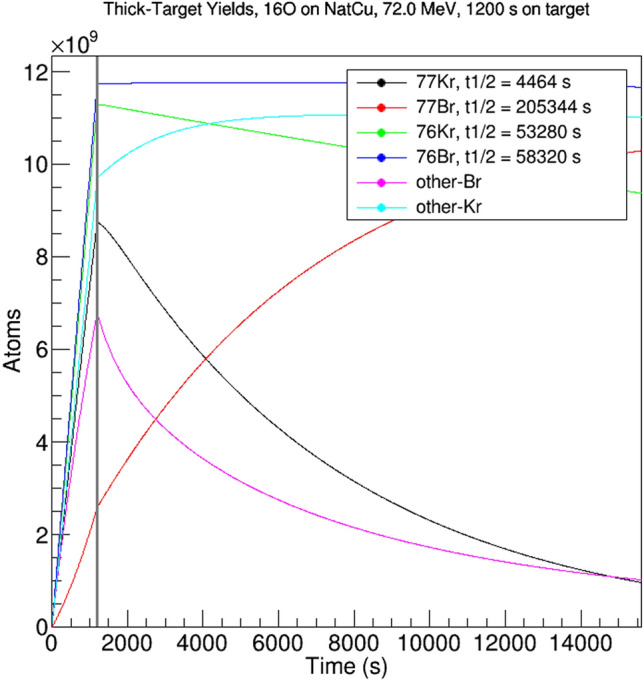
Yields, in atoms, for isotopes of interest from 1 eμA of 72 MeV ^16^O bombarding a ^nat^Cu target for 1200 s. The x-axis is seconds from start of irradiation, and the vertical grey line indicates the end of irradiation. Isotopes are labeled with their half-lives.

Theoretically, krypton and bromine isotopes can be easily and rapidly separated from one another *in vacuo* via dry distillation^5^ and differential cold-trapping. By carefully selecting when this separation occurs, it is possible to isolate specific radionuclides with high radiopurity. Figure 4 shows the calculated evolution of the number of atoms present of various relevant radionuclides as a function of time after start of irradiation, for a representative set of experimental parameters (1eμA, + 7 charge state, 72 MeV ^16^O bombarding ^nat^Cu for 20 min). At end-of-beam (EOB), all of ^76^Br and ^76/77^Kr are present in abundance, as are contaminant isotopes of both krypton and bromine. Of the krypton isotopes present, the three shortest half-lives are ^75^Kr (276 s), ^77^Kr (74.4 min) and ^76^Kr (14.8 h). ^76^Kr and ^77^Kr decay to the bromine isotopes of interest, while ^75^Kr and its daughter ^75^Br are contaminants. After 900 s, almost all (90%) of the ^75^Kr will have decayed away, with a minimal amount of ^77^Kr (13%) and ^76^Kr (1%) having decayed. At this point, bromine and krypton can be separated. The bromine will contain many isotopes but only three with a half-life longer than 97 min can be produced in this reaction: ^76^Br (16.2 h), ^77^Br (57 h) and ^79^Br (stable). After the bromine is allowed to decay for some amount of time, it will contain only ^76^Br and ^77^Br with relatively high radiopurity and a small amount (on the order of 1%) of stable ^79^Br. The exact decay time can be varied as needed to select acceptable radiopurities for a given application.

The krypton can simultaneously be allowed to decay for approximately 4 h, during which time 89% (of EOB amount) of the ^77^Kr will decay to ^77^Br, while 16% of the ^76^Kr will decay to ^76^Br and 7% of the ^79^Kr will decay to ^79^Br. Bromine and krypton can then be separated again, producing ^76^Br and ^77^Br with 51% and 48% respective radiopurity as measured by activity at 4 h EOB. The same procedure can be repeated after further decay to isolate ^76^Br from the decay of ^76^Kr. The exact decay lengths and radiopurities can again be tuned as needed for a given application.

To independently verify the statistical model predictions for these two proposed reactions mechanisms for radiobromine production, a set of complementary experiments were performed to measure the productions yield experimentally at relevant bombarding energies.

## Experimental set-up

The irradiations were performed at the Nuclear Science Laboratory (NSL) at the University of Notre Dame on the 10 MV High Voltage Engineering Corporation FN Tandem Pelletron accelerator. A Source of Negative Ions from Cesium Sputtering (SNICS) ion source was used to produce negatively charged ^28^Si or ^16^O ions. The experimental parameters used for each experiment are listed in Table 1.

**Table 1:** Experimental run parameters.

	Run 1	Run 2	Run 3
Beam	^28^Si	^16^O	^16^O
Target	^nat^Cr	^nat^Cu	^nat^Cu
Beam energy (MeV)	82.8	72.0	55.0
Terminal voltage (MV)	9.2	9.0	6.875
Charge state	+ 8	+ 7	+ 7
Runtime (min)	240	25	20
Average current (pnA)	98	111	8.4
Total charge delivered (mC)	11.2	1.2	0.070
Beamspot diameter (cm)	~ 1.0	~ 1.0	~ 1.0
SRIM range in target (μm)	11.55	22.65	16.22

**Figure 5:**
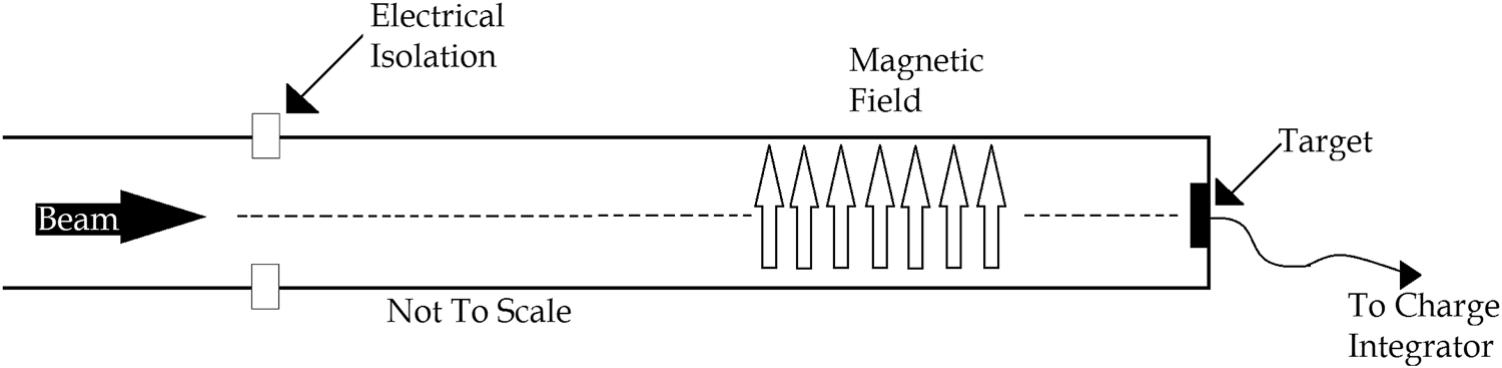
Schematic of the target mount for the ^16^O and ^28^Si irradiations. Not to scale. With the exception of the magnetic field, which is applied externally, there is radial symmetry about the dashed line. Everything downstream of the electrical isolation is electrically connected to the charge integrator.

The targets—commercially available, high-purity sputtering targets (Kurt Lesker, 99.999% Cu and 99.95% Cr, 1 inch diameter and 0.125 inch thickness)—were mounted in electrical contact with an approximately 1 foot long, closed, conducting beampipe that was itself electrically isolated from the rest of the experimental set up (see Fig. 5). A constant magnetic field was placed across this section of beampipe to direct any ejected electrons into the wall of the isolated section of beampipe, which allows for accurate charge integration and continuous current monitoring without the need for a suppression voltage. Targets were not cooled.

Activity measurements were conducted at Notre Dame via off-line gamma spectroscopy using a high-purity germanium detector. Detector efficiency was determined using ^133^Ba, ^60^Co, and ^137^Cs sources of known activity. The small (< 5%) dead-time losses were corrected by using the detector live-time in activity calculations. The target from the ^28^Si run (run 1) was counted beginning approximately 5-h after end-of-beam (EOB), with 30-min measurements continuously repeated for 10 days. Due to the high activity immediately post irradiation, the 72 MeV ^16^O irradiated copper target (run 2) was counted beginning approximately 18-h after EOB, with 30-min measurements continuously repeated for 10 days. Since much of the shorter-lived precursors would have decayed during the cooling period, reported activities (corrected to EOB) for these runs will over-predict the direct production of longer-lived products and late-produced daughters (and consequently under-predict the production of shorter-lived products and precursors) and represent net yields—that is, how much of a given radionuclide can be produced if harvesting is delayed until each decay chain of interest has settled into some long-lived species. Offline counting for the 55 MeV ^16^O run (run 3) began approximately 7 min after EOB with 1-min runs, so that over/under-attribution error does not apply to that run.

## Results

**Figure 6:**
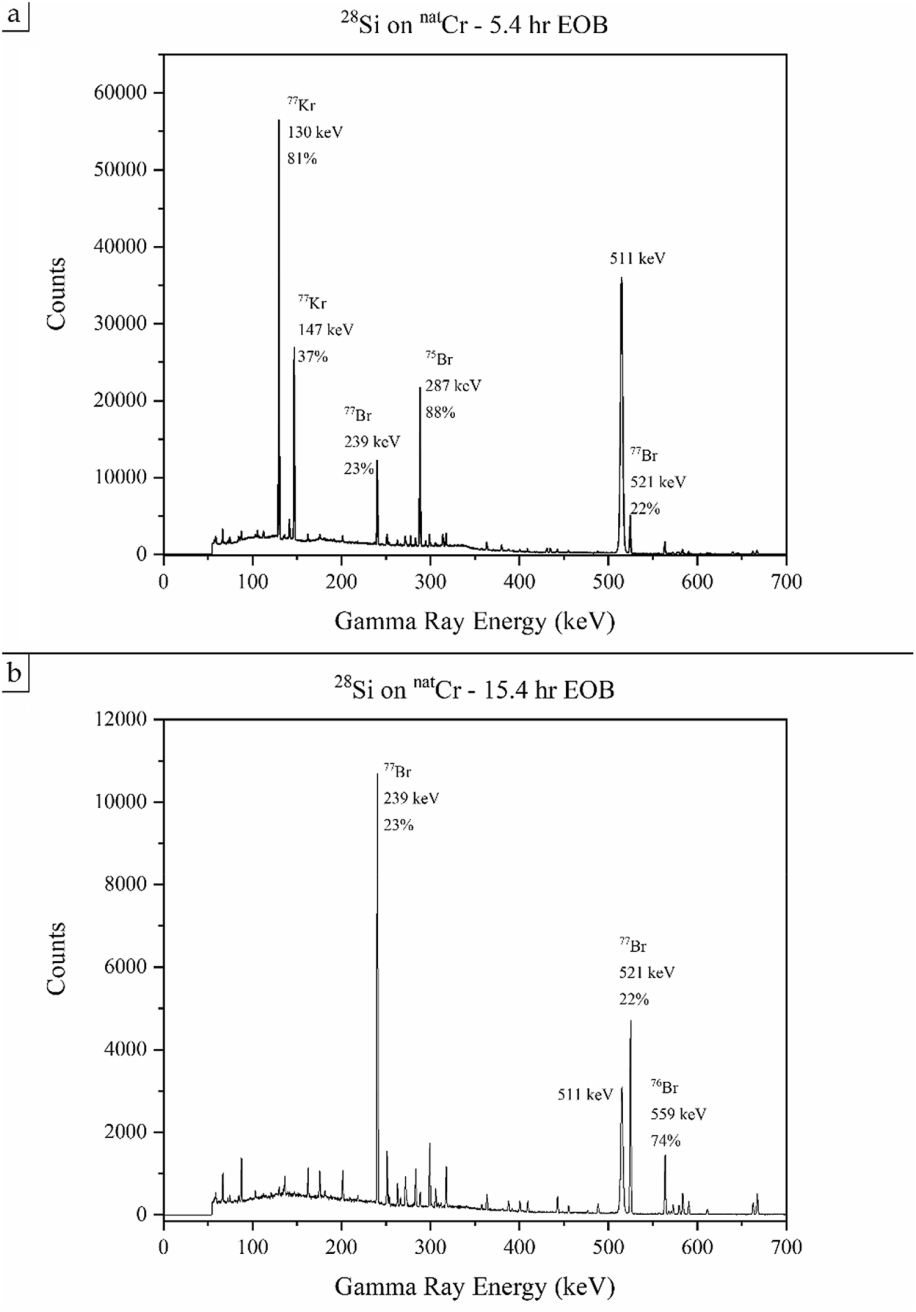
Two gamma-ray spectra resulting from the bombardment of ^28^Si on ^nat^Cr. Each spectrum was counted for 30 min, beginning 5.4 h EOB (panel **a**) or 15.4 h EOB (panel **b**). Relevant peaks are labeled with their species of origin, energy and relative intensity^16^.

Figure 6a shows a gamma spectrum measured for the ^28^Si on ^nat^Cr system 5-h after EOB. The dominant features are peaks from ^77^Kr, ^75^Br and positron annihilation. Figure 6b shows the same system after 10 further hours of decay. The contaminants and precursors have decayed away, leaving only the ^77^Br, which retains 88% of the activity it had in 6a, 83% of EOB.

**Figure 7:**
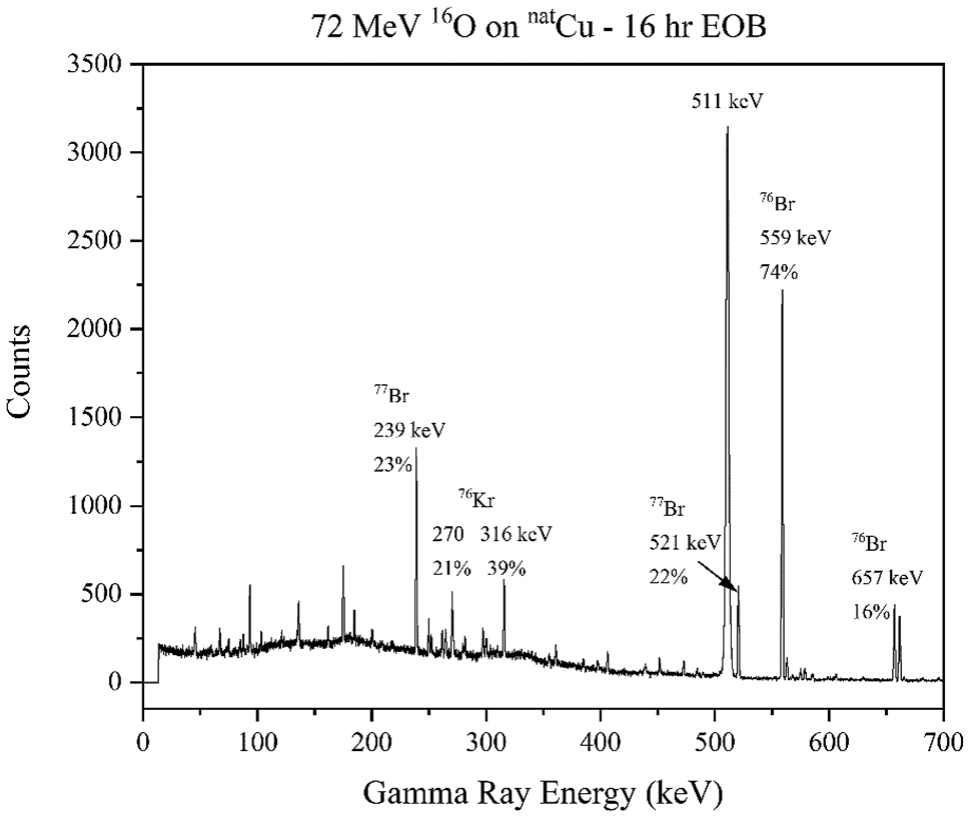
A gamma-ray spectrum resulting from the bombardment of 72 MeV ^16^O onto ^nat^Cu. The spectrum was counted for 30 min beginning 16.2 h EOB. There is significant positron annihilation, as well as strong signatures of ^77^Br, ^76^Br and ^76^Kr.

Figure 7 shows a similar scenario for the 72 MeV ^16^O on ^nat^Cu system. The first measurements were further from EOB, so the initial precursors and contaminants are not visible, but the system decays such that eventually the desired products represent the majority of the radioactivity. A complete list of radioisotopes identified by gamma spectroscopy for each run is given in the supplemental information (Table 4).

**Figure 8:**
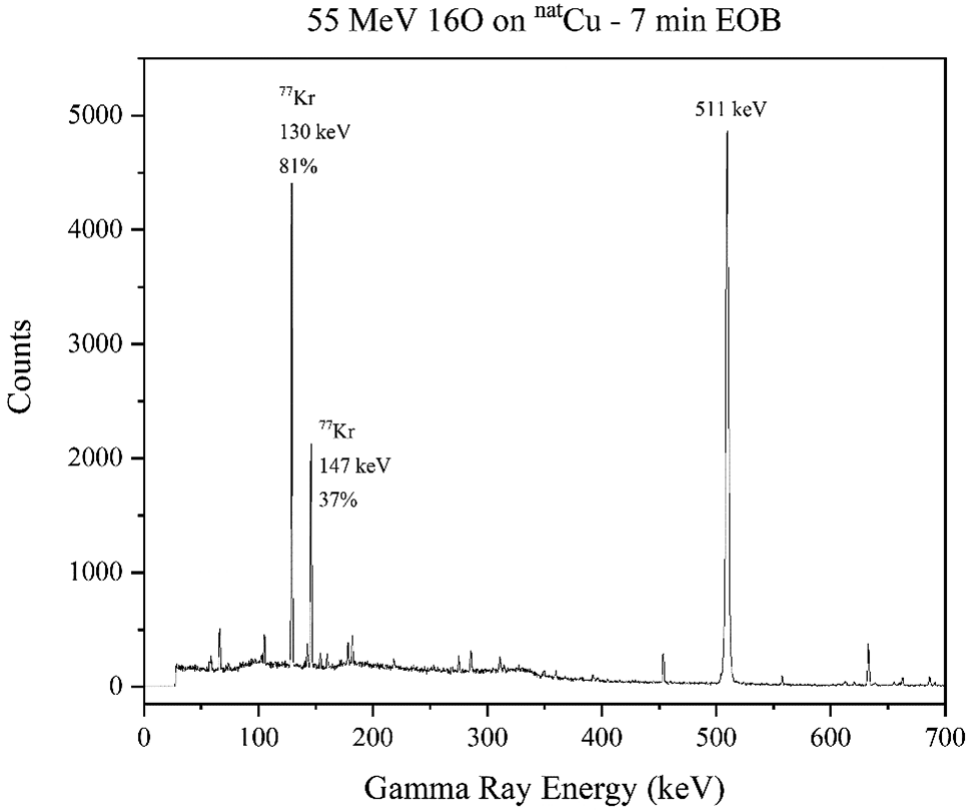
A gamma-ray spectrum resulting from the bombardment of 55 MeV ^16^O onto ^nat^Cu. The spectrum was counted for 1 min beginning 7-min EOB. There is significant positron annihilation, as well as strong signatures of ^77^Kr.

Figure 8 shows a spectrum obtained for the 55 MeV ^16^O on ^nat^Cu system, which was intentionally run at lower intensity to facilitate earlier gamma spectroscopy. The activity in the spectrum (which was only measured for 1-min, as opposed to the 30-min runs in Figs. 6 and 7) is almost entirely from ^77^Kr and positron annihilation.

Table 2 lists the runtimes, average currents, and predicted (from PACE and SRIM calculations) and measured yields for each experiment.

**Table 2:** Predicted and measured yields in kBq.

Product	82.8 MeV ^28^Si on ^nat^Cr		72.0 MeV ^16^O on ^nat^Cu			55.0 MeV ^16^O on ^nat^Cu		
	14,400 s	98 pnA	1500 s		111 pnA	1200 s		8.4 pnA
	Predicted	Measured	Predicted		Measured	Predicted		Measured
^77^Kr	805	4380 ± 170	1281^a^		< LOD^a^	44.7		51.5 ± 0.04
^77^Br	38.1	71.9 ± 15	9.21^a^/37.7^b^		5.0 ± 1.4^a^	0.33		1.0 ± 0.5
^76^Kr	17.9	31.7 ± 3.2	139		18.9 ± 4.5	1.05		0.95 ± 0.3
^76^Br	17.3	16.7 ± 1.7	134		34.6 ± 3.5	1.74		2.1 ± 1.4

^a^For the 72 MeV ^16^O experiment, counting did not begin until approximately 18 h EOB. Consequentially, any ^77^Kr produced would have decayed to a level too low to be detected prior to gamma spectroscopy beginning. The measured ^77^Br therefore represents both directly produced ^77^Br and the product of ^77^Kr decay. For both isotopes, the predicted activities are for physical yield at EOB (using the same delivered current profile as the actual irradiation). The effects of simulation error on the predicted yields are negligible compared to variations in delivered beam and are ignored.

^b^The 37.7 kBq of ^77^Br represents the predicted EOB activity that would be found if all ^77^Kr produced was allowed to decay to ^77^Br, and then an EOB activity for ^77^Br was determined by backfitting the decay from that point forward without decay-correcting for ^77^Kr feeding.

**Figure 9:**
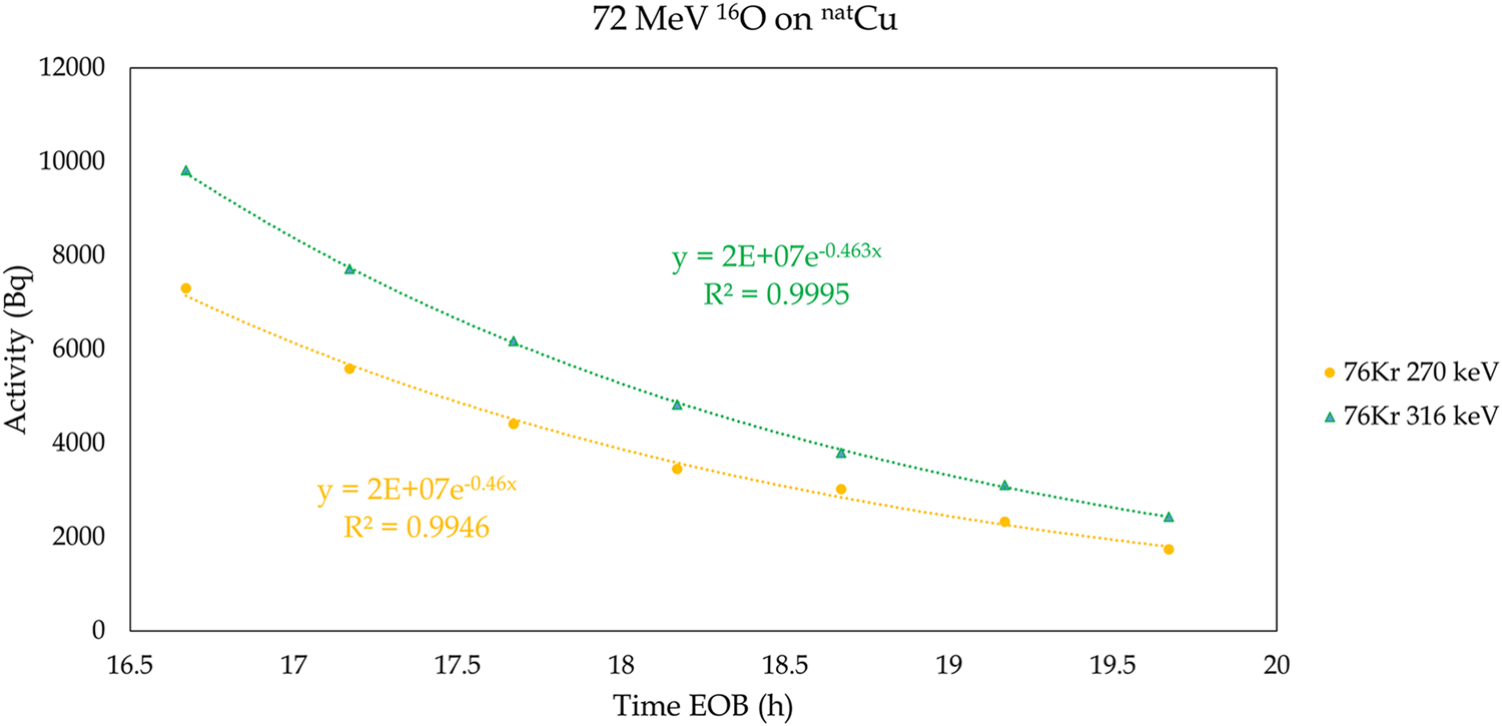
Activity vs time for two gamma signatures of ^76^Kr for the first seven spectra taken for this system. The decays are fit with simple exponentials, and the half-life of the decays is found to be 1.5 ± 0.01 h.

It is apparent from Table 2 that the measured production from the 72 MeV ^16^O run is far below what was predicted. Given the relatively good agreement between prediction and measurement for the 55 MeV ^16^O run and previous experience^11,12^ suggesting that PACE is more likely to overpredict than underpredict, it seems unlikely that the predictions were simply off to such a degree from a simple increase in bombarding energy. More detailed examinations suggest the most likely explanation is that krypton gas escaped from the target before and during gamma spectroscopy. Figure 9 shows the decay of two gamma signatures for ^76^Kr for the first seven spectra taken for this system. The time constants for the decays correspond to an experimental half-life of 1.5 h. This is off by a factor of ten from the well-measured half-life of ^76^Kr (14.8 h), so some additional physical effect on these isotopes must be in play. Since both gamma energies decay in tandem, the cause of the decreased lifetime must be affecting both peaks equally, so it cannot be independent contaminants for each energy. Since no other species have been identified which match this gamma signature, the cause cannot be spectral interference. Therefore, the most likely cause is krypton gas physically leaving the target prior to its radioactive decay. At later times (analyzed independently between *t* = 50 h and *t* = 100 h), the half-life of decay for these gamma peaks is measured to be t_1/2_ = 14.7 ± 0.2 h, which is consistent with the established lifetime for ^76^Kr. These observations are consistent with krypton which was produced near the surface of the target evaporating away, but krypton deeper in the target remaining trapped and decaying in place. In future experiments, this effect might be mitigated by cooling targets during irradiation to minimize evaporative losses.

These effects are not seen in the 55 MeV ^16^O run. This is likely a combination of more immediate gamma spectroscopy and lesser beam energy, beam current and irradiation time resulting in substantially less target heating and, by extension, less evaporative loss. SRIM calculations suggest that a 72 MeV ^16^O beam in natural copper will reduce to 55 MeV in approximately 6.4 μm, which implies that the increased krypton production would occur closer to the surface for the increased bombarding energy, and evaporation could justify the suppressed ^76^Br activity as well.

An attempt was also made to perform an alpha bombardment of an arsenic target as an additional point of comparison, but even with only a few hundred nanoamperes of alpha-beam, target degradation was sufficient to severely impact the accuracy of post-irradiation gamma spectroscopy. Because of the difficultly of reproducing measurements with such a friable target, these data were excluded from this work.

## Conclusions

These results demonstrate the utility of making proton-rich isotopes with heavy-ion reaction channels. Simple target preparation and simple radioisotope separation requirements offer advantages over current production methodologies, and do not require radiochemical purification. Although production yields are lower by 100-fold than current proton reaction methods (comparisons shown in Table 3), target robustness means that potentially higher (100-fold) beam currents can be used to achieve comparable yields to current techniques, but with more robust target material and enhanced radiopurity of the desired isotopes.

**Table 3:** Yield Comparison between proton and heavy-ion reactions (proton data from^5^).

	p on Co^77/76^Se, 16 MeV	^16^O on ^nat^Cu, 55 MeV^a^	p on Co^77/76^Se, 16 MeV^b^	^16^O on ^nat^Cu, 55 MeV^b^	^16^O on ^nat^Cu, 72 MeV^b^
	Physical yield (MBq∙μA^−1^∙h^−1^)	Physical yield (MBq∙μA^−1^∙h^−1^)	3-h irradiation (GBq)	3-h irradiation (GBq∙pμA^−1^)	3-h irradiation (GBq∙pμA^−1^)
^77^Br	23	0.686	2.8	0.0012	0.0022
^76^Br	88	0.681	10.6	0.0016	0.0086

^a^To account for indirect production, physical yields for the heavy-ion reaction are given for 4 h EOB, to allow ~ 90% of the ^77^Kr to decay to ^77^Br.

^b^Since 3-h irradiations were not performed, the absolute yields are scaled from physical yield (accounting for saturation and decay effects) and reported per-particle-microamp of beam delivered (the proton data assumed a 40 μA max current for the entirety of the run). The 55 MeV data are based on data reported in this work. The 72 MeV data are a prediction from PACE calculations, since the measured data were likely depleted by krypton evaporation.

On a per-incident-particle basis, yields from the heavy-ion reactions are suppressed by approximately two orders of magnitude, but much of this shortfall may be offset with increased beam current, with significantly easier targetry and purification processes. The short range (< 25 μm) of the heavy-ion projectiles means that all products are near to the target surface and should be recoverable without dissolving the target. With enriched targeted materials (shown in supplemental data), heavy ion reactions also offer routes to pure ^76^Br and ^77^Br.

Targets made from natural copper are also reusable, meaning that targets can be cycled between irradiation and extraction (*in vacuo*) for routine production. With the observation of evaporative losses from the room temperature target, the possibility of continuous irradiation and on-line separation emerges. Krypton evaporating from the target could be pumped away and cold-trapped, and harvesting could potentially take place without ever having to stop irradiation, access the target or perform separation chemistry with hot targets. With modern ion sources capable of delivering abundant silicon or oxygen beams (see^17^, or a commercially available source, e.g.^18^) it is feasible to construct a dedicated production facility that could routinely produce amounts of radiobromine suitable for clinical use. Such a facility may also be able to make large-scale production of several other proton-rich radioisotopes economically feasible.

## Supplementary Information

Supplementary Information 1.
