# Supplementary material for: Heavy-ion production of 77Br and 76Br
Source: Sci Rep. 2021 Aug 3;11:15749. doi: 10.1038/s41598-021-94922-x (PMC8333326; doi:10.1038/s41598-021-94922-x)
Supplement: Supplementary file 1 — Supplementary Information 1. [file 41598_2021_94922_MOESM1_ESM.docx]

**Heavy-Ion Production of ^77^Br and ^76^Br**

Sean R. McGuinness^1+^, John T. Wilkinson^1^, and Graham F. Peaslee^1^

^+^Corresponding Author, smcguinn@nd.edu

^1^Department of Physics, University of Notre Dame, Notre Dame, IN 46556

# Supplemental


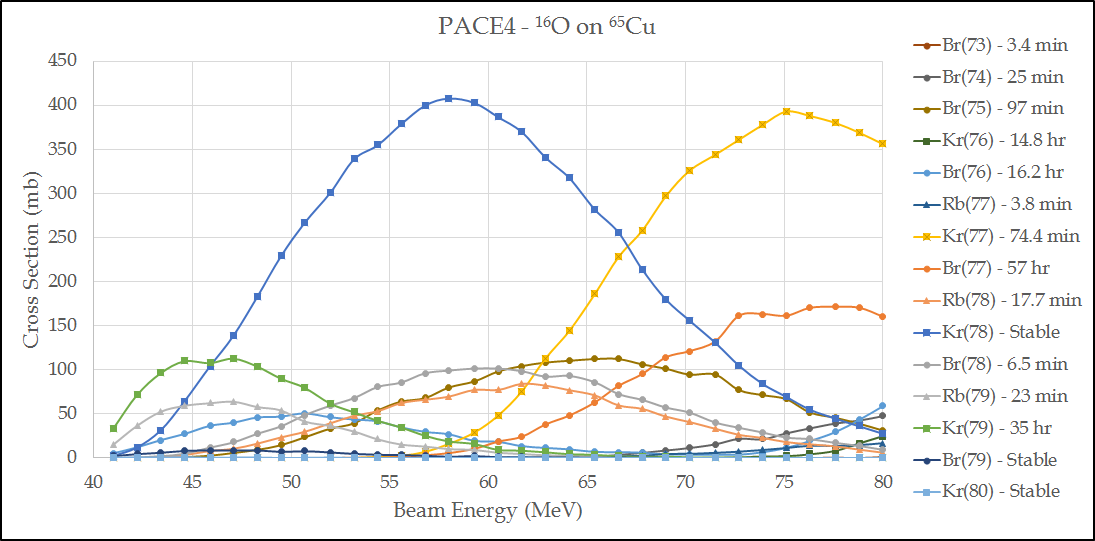


Figure 10 - PACE4 predicted cross sections for ^16^O on ^65^Cu.


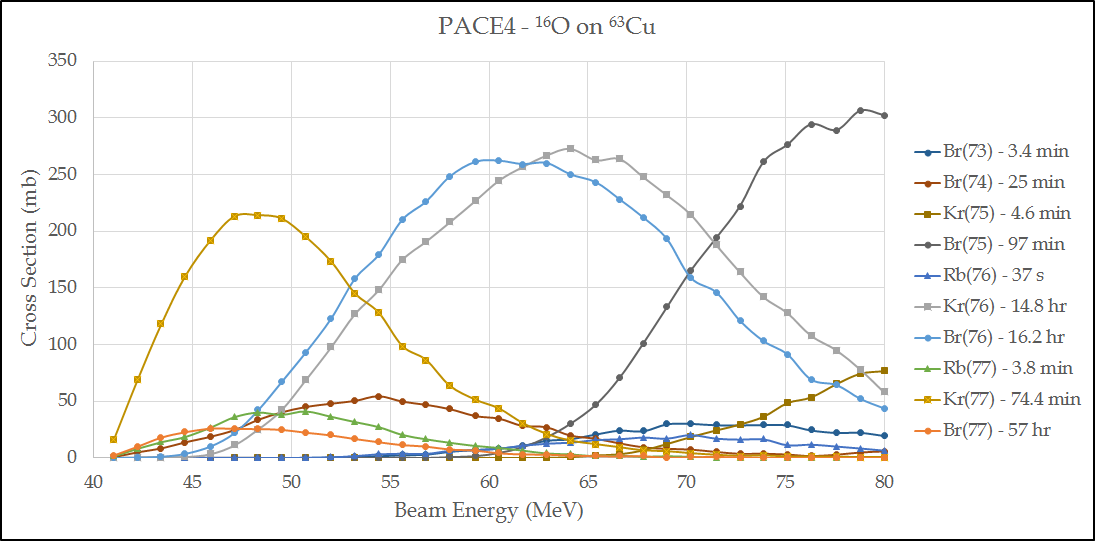


Figure 11 - PACE4 predicted cross sections for ^16^O on ^63^Cu.

Table 4 - Residual Gamma Peaks

| Radionuclide | Energies (keV) | Runs |
| --- | --- | --- |
| ^77/77m^Br | 105.9, 249.8, 281.7, 297.2 | 1,2 |
| ^76^Kr | 45.5, 252.0, 406.5 | 2 |
| ^75^Se | 136, 264.7, 400.7 | 1,2 |
| ^67^Cu | 93.3, 184.6 | 2 |
| ^78/78m^Rb | 455.0, 664.4 | 1,2,3 |
| ^79^Kr | 261.3, 306.1 | 1,2 |
| ^77^Rb | 66.5 | 1,2,3 |
| ^74^Br | 634.8 | 3 |
| ^75^Br | 286.5 | 3 |
| ^76^Br | 559.1 | 3 |
| ^77^Kr | 311.9 | 3 |
| ^77m^Se? | 161.9 | 1 |
| ^137^CS | 661.7 | 1,2,3 |
| ^224^Th? | 409.8 | 1,2 |
| Atomic Pb | 88.0 | 1 |
| Unknown | 176 | 1,2,3 |
| Unknown | 201 | 1,2 |
| Unknown | 387.7 | 1 |
| Unknown | 443 | 1,2 |
| Unknown | 564 | 1 |
